# Clusterin Attenuates Hepatic Fibrosis by Inhibiting Hepatic Stellate Cell Activation and Downregulating the Smad3 Signaling Pathway

**DOI:** 10.3390/cells8111442

**Published:** 2019-11-14

**Authors:** Hye-Young Seo, So-Hee Lee, Ji-Ha Lee, Yu Na Kang, Young-Keun Choi, Jae Seok Hwang, Keun-Gyu Park, Byoung Kuk Jang, Mi Kyung Kim

**Affiliations:** 1 Department of Internal Medicine, Keimyung University School of Medicine, Daegu 42601, Korea; seo568@hanmail.net (H.-Y.S.); jy16162727@naver.com (S.-H.L.); jihain10@gmail.com (J.-H.L.); gastro@dsmc.or.kr (J.S.H.); 2 Institute for Medical Science, Keimyung University School of Medicine, Daegu 42601, Korea; 3 Department of Pathology, Keimyung University School of Medicine, Daegu 42601, Korea; Yunakang@dsmc.or.kr; 4 Laboratory Animal Resource Center, Korea Research Institute of Bioscience and Biotechnology (KRIBB), Daejeon 34141, Korea; keun46@kribb.re.kr; 5 Department of Internal Medicine, School of Medicine, Kyungpook National University, Daegu 41944, Korea; kpark@knu.ac.kr

**Keywords:** clusterin, hepatic stellate cells, hepatic fibrosis, Smad3

## Abstract

Clusterin is a glycoprotein that is expressed in most human tissues and found in body fluids. In our previous studies we demonstrated that clusterin has a protective effect against hepatic lipid accumulation and renal fibrosis; however, the role of clusterin in hepatic fibrosis is unknown. Here, we examined whether clusterin had protective effects against hepatic fibrosis using in vitro and in vivo models. Clusterin was upregulated in the livers of human cirrhotic patients and in thioacetamide (TAA)-induced and bile duct ligation mouse models of liver fibrosis. Loss and overexpression of clusterin promoted and attenuated hepatic fibrosis after TAA injection, respectively. In addition, we found that clusterin attenuates hepatic fibrosis by inhibiting the activation of hepatic stellate cells and Smad3 signaling pathways. Thus, clusterin plays an important role in hepatic fibrosis.

## 1. Introduction

Hepatic fibrosis is the result of chronic liver injury caused by conditions such as viral infection, alcohol toxicity, and metabolic overload [1,2]. Fibrosis is a common pathological process for most liver diseases, causing cirrhosis and hepatocellular carcinoma in a significant number of patients [3]. Currently, liver fibrosis therapy involves treatment of the underlying diseases, such as antiviral therapy for chronic hepatitis B and hepatitis C [4]. There are no antifibrotic therapies available at present, but various studies have revealed the pathogenesis of liver fibrosis, and a number of therapeutic drugs are currently being developed [5,6].

Clusterin (apolipoprotein J), a glycoprotein that is expressed in most human tissues and found in body fluids [7], is constitutively synthesized and plays a role in several biological events, such as programmed cell death, lipid transport, complement regulation, and barrier cytoprotection [8,9,10]. The expression level of clusterin is increased significantly in various pathological conditions, such as neurodegenerative disease, kidney injury, and atherosclerosis [8,11,12]. In our previous study, we showed that clusterin reduces hepatic lipid accumulation by inhibiting hepatic sterol regulatory binding protein-1c [13]. In addition, it was recently reported that overexpression of hepatocyte-specific clusterin prevents steatohepatitis [14]. These results indicate that clusterin regulates lipid metabolism to improve fatty liver and steatohepatitis; however, its potential role in improving liver fibrosis is still unknown.

In this study, we investigated the effect of clusterin on liver fibrosis in mice and hepatic stellate cells (HSCs) using a thioacetamide (TAA)-induced liver fibrosis model.

## 2. Materials and Methods

### 2.1. Chemicals

TAA was purchased from Sigma-Aldrich (St. Louis, MO, USA), and recombinant human transforming growth factor beta (TGF-β), 5 ng/mL was purchased from R&D Systems (Minneapolis, MN, USA). The anti-clusterin (sc-6420) and anti-phospho-Smad2/3 (sc-11769) antibodies were purchased from Santa Cruz Biotechnology (Dallas, TX, USA). The anti-collagen antibody (ab-34710) was purchased from Abcam (Cambridge, UK), and the anti-αSMA antibody (A2547) was purchased from Sigma-Aldrich. The anti-GAPDH (cs-2118), anti-phospho-Smad3 (Ser423/425), and anti-Smad3 (cs-9520) antibodies were purchased from Cell Signaling Technology (Beverly, MA, USA).

### 2.2. Isolation of Primary HSCs

HSCs were isolated from C57BL/6 (wild type) and clusterin knock out (KO) mice by perfusing the liver through the inferior vena cava. The liver was perfused with EGTA buffer (136.89 mmol/L NaCl, 5.37 mmol/L KCl, 0.64 mmol/L NaH2PO4.H2O, 0.85 mmol/L Na2HPO4, 9.99 mmol/L HEPES, 4.17 mmol/L NaHCO3, 0.5 mmol/L EGTA, and 5 mmol/L glucose (pH 7.35–7.4)) at a rate of 5 mL/min for 2 min, followed by enzyme buffer (136.89 mmol/L NaCl, 5.37 mmol/L KCl, 0.64 mmol/L NaH2PO4.H2O, 0.85 mmol/L Na2HPO4, 9.99 mmol/L HEPES, 4.17 mmol/L NaHCO3, and 3.81 mmol/L CaCl2.2H2O (pH 7.35–7.4)) containing 0.4 mg/mL pronase (Roche Diagnostics, Indianapolis, IN, USA) at a rate of 5 mL/min for 5 min, and then enzyme buffer containing 0.193 U/mg collagenase (Roche Diagnostics) at a rate of 5 mL/min for 7 min. After perfusion, the liver was shaken for 25 min at 37 °C, filtered through a 70 µm nylon mesh, and centrifuged at 580× *g* for 10 min at 4 °C. Pelleted HSCs were resuspended in Gey’s Balanced Salt Solution (GBSS) (Sigma-Aldrich), gently overlaid with a gradient of Cell-OptiPrep™ (Sigma-Aldrich) prepared with GBSS using a pipette, and then centrifuged at 1380× *g* for 17 min at 4 °C without braking. HSCs present in a thin white layer at the interface between Cell-OptiPrep™ and GBSS were harvested and washed with Hank’s Balanced Salt Solution. The cells were plated in DMEM (Gibco-BRL, Grand Island, NY, USA) containing 10% fetal bovine serum (FBS). The medium was changed every 2 days.

### 2.3. Animals

In vivo experiments were conducted using 8- to 9-week-old male C57BL/6 mice (Samtako, Osan, Korea). To generate clusterin KO mice on the C57BL/6 genetic background, clusterin-deficient mice that were originally generated using a Swiss black genetic background were backcrossed onto the C57BL/6 strain for at least seven generations. All experiments were approved by the Institutional Animal Care and Use Committee of Keimyung University (KM-2017-32R1). All animal procedures were carried out in accordance with institutional guidelines for animal research.

#### 2.3.1. Animal Experiments 1 (TAA-Induced Liver Fibrosis Model)

Liver fibrosis was induced by intraperitoneal injection of wild type (n = 5) and clusterin KO mice (n = 7) with TAA (200 mg/kg body weight), three times per week for 8 weeks. In the in vivo infection study, mice injected with TAA for 7 weeks were divided into two groups and injected via the tail vein with adenoviruses expressing GFP (Ad-GFP, n = 5) or clusterin (Ad-Clu, n = 5) (1 × 10^9^ pfu per 200 µL). Animals were euthanized after 1 week. 

#### 2.3.2. Animal Experiments 2 (Bile Duct Ligation (BDL)-Induced Liver Fibrosis Model)

For bile duct ligation (BDL), mice were randomly divided into three experimental groups: sham-operated control (n = 5), BDL 3 days (n = 5), and BDL 7 days (n = 5). The BDL animals were anesthetized with pentobarbital (50 mg/kg), and a midline laparotomy was performed. The common bile duct was doubly ligated with 5-0 silk and then transected between the two ligatures. The sham operation was identical but was performed without ligation.

#### 2.3.3. Animal Experiments 3 (High-Fat Diet Fed Mice)

Mice were fed a high-fat diet that provided 60% of the calories as fat (D12492; Research Diets, New Brunswick, NJ, USA). After 12 months, the animals were killed and liver samples were collected (chow diet n = 6, HFD n = 6).

### 2.4. Cell Culture

The LX2 human hepatic stellate cell line was a kind gift from Kr. Jeong (Korea Advanced Institute of Science and Technology, Daejeon, Korea). LX2 cells were cultured in 5% CO_2_/95% air at 37 °C in DMEM (Gibco-BRL) supplemented with 10% FBS (Hyclone, Logan, UT, USA) and antibiotics (1% penicillin/streptomycin). The cells were serum starved in medium containing 0.5% FBS and then infected with viruses (Ad-GFP = 100 moi; Ad-Clu = 50 moi, 100 moi) [13,15]. After 2 h the medium was changed and treated with TGF-β, 5 ng/mL for 24 h.

### 2.5. Generation of Recombinant Adenovirus

The cDNA encoding rat clusterin was inserted into the pAd-Track-CMV shuttle vector. To produce the recombinant adenoviral plasmid, the resultant shuttle vector was electroporated into BJ5138 cells containing the AdEasy adenoviral vector. The recombinant adenoviral plasmids were transfected, and adenoviruses expressing clusterin were amplified in human embryonic kidney-293 cells and purified using CsCl density centrifugation (Sigma-Aldrich). The viruses were collected and desalted, and the titers were determined using the Adeno-X Rapid Titer Kit (BD Bioscience, San Jose, CA, USA).

### 2.6. Quantitative Real-Time RT-PCR

Total RNA was isolated from cells using TRIzol reagent (Life Technologies, Grand Island, NY, USA). Reverse transcription was performed using the Maxima First Strand cDNA Synthesis Kit (Thermo Scientific, Rockford, IL, USA). Quantitative real-time RT-PCR was performed using a SYBR Green Master Mix kit (Roche Diagnostics) and the LightCycler 480 Real-Time PCR System (Roche Diagnostics). PCR parameters were as follows: 95 °C for 30 s, 60 °C for 10 s and 72 °C for 15 s. The primer sequences were as follows: mouse collagen: forward, 5′-GCCTTGGAGGAAACTTTGCTT-3′ and reverse, 5′-GCACGGAAACTCCAGCTGAT-3′; mouse αSMA: forward, 5′-CAGGCTGTGCTGTCCCTCTA-3′ and reverse, 5′-CGGCAGTAGTCACGAAGGAA-3′; mouse PAI-1 (plasminogen activator inhibitor-1): forward, 5′-AAATCCCACACAGCCCATCA-3′ and reverse, 5′-GGACCACCTGCTGAAACACTTT-3′; and mouse GAPDH: forward, 5′-ACGACCCCTTCATTGACCTC-3′ and reverse, 5′-ATGATGACCCTTTTGGCTCC-3′. Expression of GAPDH mRNA was used as an internal control.

### 2.7. Western Blotting

LX2 cells were treated with TGF-β (5 ng/mL) and infected with adenoviral vectors expressing clusterin. The cells were harvested in lysis buffer (50 mmol/L Tris-HCl (pH 8.0), 150 mmol/L NaCl, 1 mmol/L EDTA, 1% Triton X-100, and 0.25% Na-deoxycholate) containing protease/phosphatase inhibitors (Inhibitor Cocktail solution, genDEPOT) and dithiothreitol (0.5 mM). Nuclear extracts were isolated from the cells using the NucBusterTM Protein Extraction Kit (Calbiochem, La Jolla, CA, USA), according to the manufacturer’s instructions. Proteins were resolved by SDS-PAGE and then transferred electrophoretically to a polyvinyl difluoride membrane (Millipore, Burlington, MA, USA). The membranes were sequentially incubated in blocking buffer (5% skimmed milk prepared in Tris-buffered saline containing 0.1% Tween 20), primary antibodies, and appropriate horseradish peroxidase-conjugated secondary antibodies. Signals were visualized using the Clarity™ Western ECL Substrate Kit (Bio-Rad, Richmond, CA, USA). The membrane was re-probed with an anti-GAPDH antibody to verify that an equal amount of protein had been loaded in each lane. Signal intensities were quantitated by densitometry using ImageJ software (version 1.52a) (NIH, Bethesda, MD, USA).

### 2.8. Immunohistochemical Analysis

Livers were isolated from mice, fixed in 4% formaldehyde, and then paraffin-embedded. Histochemical staining was performed using hematoxylin and eosin, Sirius red, and trichrome stains. Immunohistochemical staining was performed by incubation with primary antibodies against collagen (1:500), αSMA (1:500), phospho-Smad2/3 (1:300), and clusterin (1:200), followed by horseradish peroxidase-conjugated anti-mouse, anti-rabbit (Dako, Glostrup, Denmark), and anti-goat (Abcam) IgG secondary antibodies. All data were normalized against the equivalent data in mice fed chow (control). Immunostaining was quantified using ImageJ software (ImageJ software, 1.52a National Institutes of Health, Bethesda, MD).

### 2.9. Patients and Specimens

Liver tissues were obtained from 11 patients with intrahepatic duct stones (non-fibrotic samples) and 14 patients with cirrhosis who underwent hepatectomy at Keimyung University, Dongsan Medical Center, between January 2002 and December 2018. The study was conducted in accordance with the Declaration of Helsinki, and the protocol was reviewed and approved by the Institutional Review Board of Keimyung University Dongsan Hospital (IRB No. 2019-07-061). Formalin-fixed and paraffin-embedded tissue blocks were selected for tissue microarray sampling. A puncher tip (5.0 mm in diameter) was used to punch out representative non-fibrotic and cirrhotic areas. Three cores per case were used to construct a tissue microarray. Immunohistochemical staining was performed using a rabbit anti-Src polyclonal antibody (Abcam, Cambridge, MA, USA) and an automatic staining device (Benchmark XT; Ventana Medical Systems, Mountain View, CA, USA) in accordance with the manufacturers’ protocols.

### 2.10. Statistical Analysis

Data are expressed as the mean ± SEM. Statistical analyses were performed using a one-way ANOVA analysis with Duncan’s test or a two-tailed Student’s *t*-test. *p* < 0.05 was considered statistically significant. All experiments were performed at least three times.

## 3. Results

### 3.1. Clusterin Expression Is Elevated in the Fibrotic Liver

**Figure 1:**
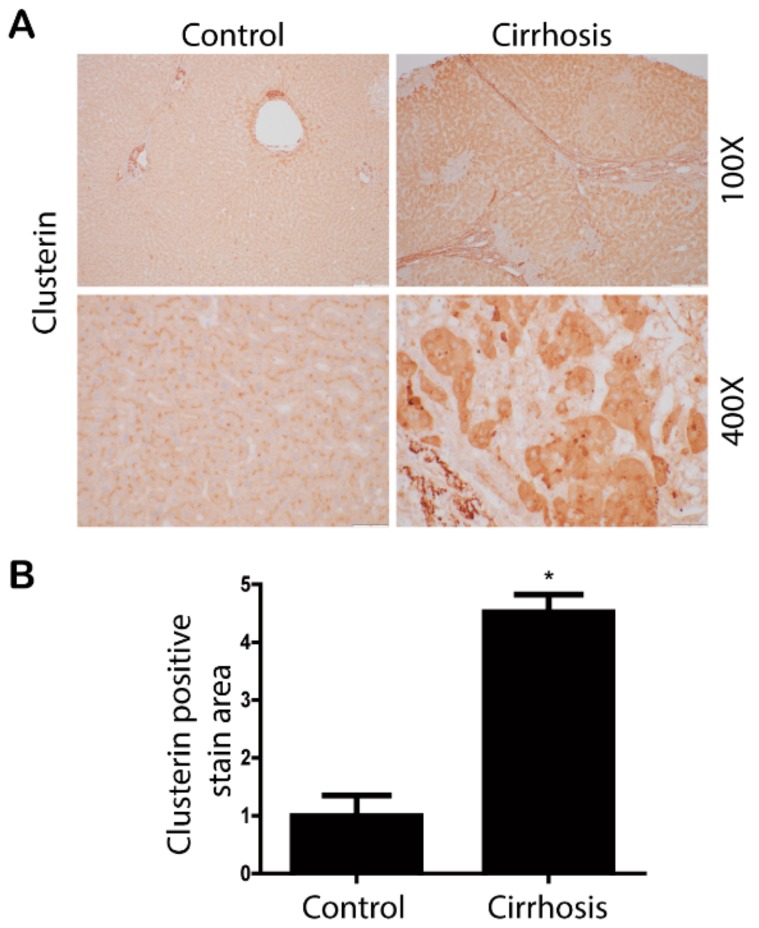
Clusterin is upregulated in the cirrhotic liver. (**A**) Representative images of immunohistochemical staining of clusterin in control and cirrhotic human liver samples. (**B**) Areas of positive clusterin immunostaining were quantified by computer-based morphometric analysis. All morphometric data of cirrhotic livers were normalized against those of the control, and the data in all bar graphs are expressed as fold increases relative to the control. Data in the bar graph are means ± SEM. * *p* < 0.05 compared with the control.

**Figure 2:**
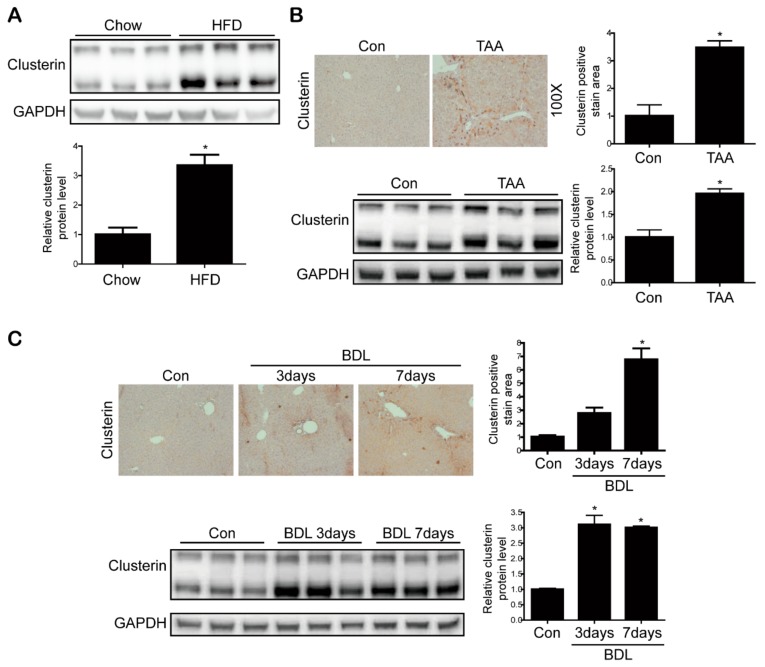
Clusterin is involved in hepatic fibrosis. (**A**) Representative western blot analyses of clusterin expression in the livers of mice fed a high-fat diet (HFD) or chow (control) for 1 year. Data in the bar graph are means ± SEM. * *p* < 0.05 compared with the control. (**B**,**C**) Representative Immunohistochemistry and western blot analysis of clusterin expression in the mouse liver after TAA-(**B**) and BDL (**C**). Areas of positive clusterin immunostaining were quantified by computer-based morphometric analysis. All morphometric data of mouse livers obtained after TAA and BDL were normalized against those of the control, and the data in all bar graphs are expressed as fold increases relative to the control. Data in the bar graph are means ± SEM. * *p* < 0.05 compared with the control.

First, we investigated whether the expression level of clusterin is altered in the fibrotic liver. Immunohistochemical analyses of liver tissues from 11 patients with control samples and 14 patients with cirrhosis revealed that the expression level of clusterin was upregulated in the cirrhotic condition (Figure 1). Next, we confirmed the expression of clusterin in the fibrotic animal models. In the livers of non-alcoholic fatty liver disease mice fed a high-fat diet for one year, clusterin expression was higher than that in control mice (Figure 2A). Subsequently, we induced hepatic fibrosis in mice by intraperitoneal injection of TAA for eight weeks. Immunohistochemical staining and western blotting showed that clusterin expression was higher in the livers of TAA-injected mice than those of control mice (Figure 2B). In addition, clusterin was highly expressed in mice that underwent BDL, another model of liver fibrosis (Figure 2C). Overall, these data suggest that clusterin plays an important role in hepatic fibrosis.

### 3.2. Loss of Clusterin Promotes Hepatic Fibrosis

**Figure 3:**
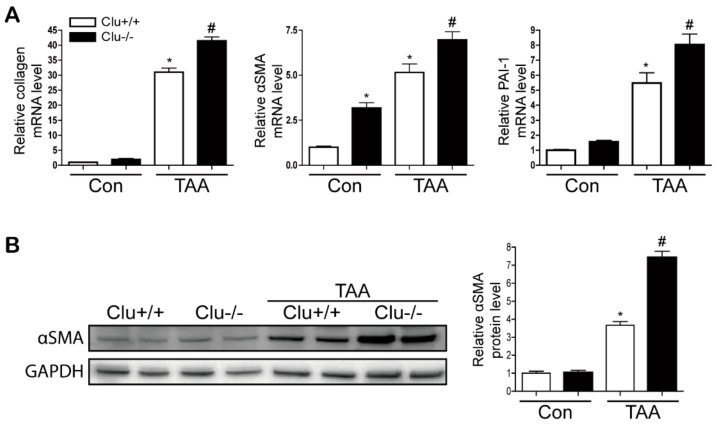
Loss of clusterin increases the expression of type I collagen, αSMA, and PAI-1 after TAA injection. (**A**) Representative real-time RT-PCR analyses of type I collagen, αSMA, and PAI-1 mRNA levels in the livers of control (Con) and TAA-injected wild-type (Clu+/+; white bars) and clusterin KO (Clu−/−; black bars) mice. * *p* < 0.05 compared with Clu+/+, ^#^ *p* < 0.05 compared with Clu+/+ after TAA injection. (**B**) Representative western blot analysis of αSMA expression in the livers of wild-type (Clu+/+) and clusterin KO (Clu−/−) mice after TAA injection. Data in the graph are represented as the mean ± SEM. * *p* < 0.05 compared with Clu+/+, ^#^ *p* < 0.05 compared with Clu+/+ after TAA injection.

**Figure 4:**
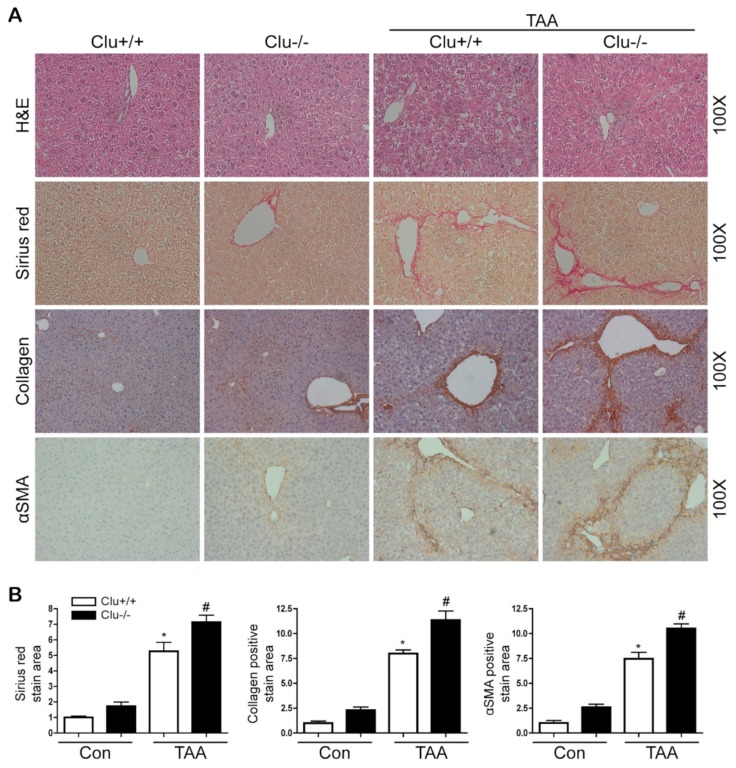
Loss of clusterin accelerates hepatic fibrosis after TAA injection. (**A**,**B**) Representative images of hematoxylin and eosin (H&E), Sirius red, and immunohistochemical staining of liver tissue sections from wild-type (Clu+/+) and clusterin KO (Clu−/−) mice after TAA injection. Areas of positive staining with Sirius red and antibodies were quantified by computer-based morphometric analysis. All morphometric data were normalized against the corresponding values in Clu+/+ mice, and data in all graphs are expressed as fold-increases relative to the Clu+/+ mice. Data are represented as the mean ± SEM of five random fields from each liver. Original magnification, ×100. * *p* < 0.05 compared with Clu+/+, ^#^ *p* < 0.05 compared with Clu+/+ after TAA injection.

Next, the effect of clusterin on hepatic fibrosis was examined using clusterin KO mice. TAA injection promoted hepatic fibrosis in wild-type mice, and the loss of clusterin aggravated the fibrotic condition further. Levels of the mRNAs encoding type I collagen, αSMA, and PAI-1 were increased after TAA injection, and their expression levels were higher in the livers of TAA-injected clusterin KO mice than in those of TAA-injected wild-type mice. In addition, αSMA and PAI-1 expression was higher in the TAA-injected clusterin KO mice than in the TAA-injected wild-type mice (Figure 3 and Supplementary Figure S1). Sirius red staining revealed that fibrosis was higher in clusterin KO mice than in wild-type mice. In addition, immunohistochemical staining revealed that the expression levels of type I collagen and αSMA were higher in clusterin KO mice than in wild-type mice (Figure 4). These data suggest that loss of clusterin promotes hepatic fibrosis.

### 3.3. Clusterin Ameliorates TAA-Induced Hepatic Fibrosis

**Figure 5:**
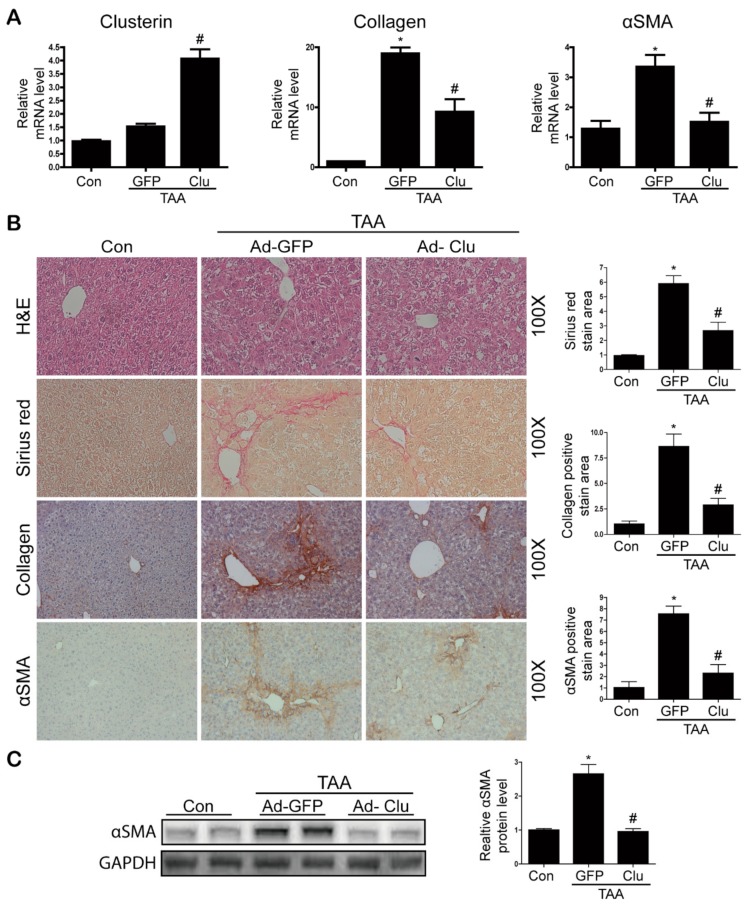
Adenovirus-mediated overexpression of clusterin ameliorates hepatic fibrosis after TAA injection. (**A**) Representative real-time RT-PCR analyses of clusterin, type I collagen, and αSMA mRNA levels in the livers of mice infected with Ad-GFP or Ad-Clu. * *p* < 0.05 compared with the control (Con), ^#^ *p* < 0.05 compared with Ad-GFP after TAA injection. (**B**) Representative images of hematoxylin and eosin (H&E), Sirius red, and immunohistochemical staining of liver sections from control and TAA-injected mice expressing Ad-GFP or Ad-Clu. Areas of positive staining with Sirius red and the antibodies were quantified by computer-based morphometric analysis. All morphometric data were normalized against the corresponding values in control samples, and data in all graphs are expressed as fold-increases relative to the control. Data are represented as the mean ± SEM of five random fields from each liver. Original magnification, ×100. * *p* < 0.05 compared with the control (Con), ^#^ *p* < 0.05 compared with Ad-GFP after TAA injection. (**C**) Representative western blot analysis of αSMA expression in TAA-injected mice expressing Ad-Clu. Data in the graph are represented as the mean ± SEM. * *p* < 0.05 compared with the control (Con), ^#^ *p* < 0.05 compared with Ad-GFP after TAA injection.

To evaluate the inhibitory effect of clusterin on TAA-induced hepatic fibrosis further, mice were infected with a clusterin-containing adenovirus (Ad-Clu) via the tail vein, and clusterin gene expression was detected by real-time RT-PCR. Sirius staining showed that Ad-Clu significantly reduced fibrosis. In addition, immunohistochemical staining demonstrated that Ad-Clu infection attenuated the TAA-induced increases in the expression levels of type I collagen and αSMA (Figure 5A). Subsequently, we confirmed the effect of clusterin on liver fibrosis using RT-PCR and western blot analyses. Infection of mice with Ad-Clu significantly reduced TAA-induced expression of the type I collagen and αSMA mRNAs (Figure 5B), as well as of the αSMA protein (Figure 5C).

### 3.4. Clusterin Ameliorates HSC Activation

**Figure 6:**
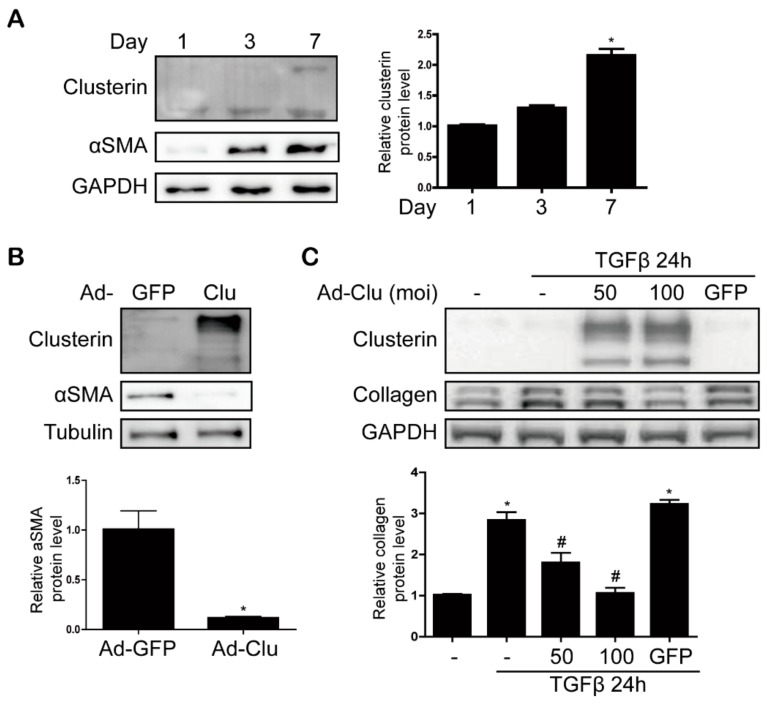
Clusterin inhibits αSMA in primary HSC and collagen expression in LX2 cells. (**A**) Representative western blot analysis of clusterin during HSC activation. Data in the graph are represented as the mean ± SEM of three independent measurements. * *p* < 0.05 compared with Day 1. (**B**) Western blot analysis of αSMA expression in cultured HSCs. Primary HSCs were infected with Ad-Clu and harvested 24 h later. Data in the graph are represented as the mean ± SEM of three independent measurements. * *p* < 0.05 compared with Ad-GFP. (**C**) Western blot analysis showing the effect of clusterin on TGF-β-induced collagen expression in LX2 cells. Data in the graph are represented as the mean ± SEM of four independent measurements. * *p* < 0.05 compared with the control, ^#^ *p* < 0.05 compared with TGF-β.

Because activated HSCs contribute to the progression of liver fibrosis, we cultured isolated quiescent HSCs for seven days to become activated HSCs and confirmed the expression of αSMA. Clusterin expression also increased during HSC activation (Figure 6A). To confirm the effects of clusterin on HSC activation, we infected primary HSCs with Ad-Clu. As shown in Figure 6B, overexpression of clusterin inhibited αSMA expression at three days in primary HSCs from wild-type mice. In HSC activated on day 8, clusterin inhibited collagen expression (Supplementary Figure S2). In addition, clusterin inhibited TGF-β-stimulated collagen and fibronectin expression in LX2 cells (Figure 6C and Supplementary Figure S3). These results suggest that clusterin ameliorates HSC activation.

### 3.5. Clusterin Inhibits Smad3 Phosphorylation

**Figure 7:**
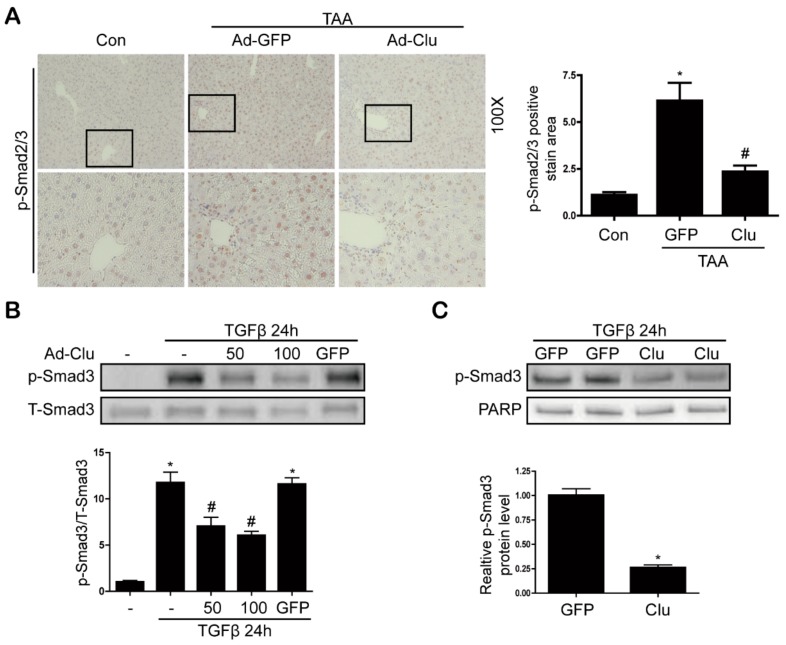
Clusterin inhibits TAA- or TGF-β-induced nuclear translocation of Smad2 and Smad3. (**A**) Representative images of immunohistochemical staining of phospho-Smad2/3 in liver sections of mice injected with or without TAA, and infected with Ad-GFP or Ad-Clu. Data in the bar graph are means ± SEM. * *p* < 0.05 compared with control (Con). ^#^ *p* < 0.05 compared with TAA-injection (Ad-GFP). (**B**) Western blot analyses of the effects of clusterin on TGF-β-induced phospho-Smad3 expression in whole cell extracts of LX2 cells. Data in the graph are represented as the mean ± SEM of four independent measurements. * *p* < 0.05 compared with the control, ^#^ *p* < 0.05 compared with TGF-β. (**C**) Western blot analyses of nuclear extracts of LX2 cells showing the effects of clusterin on TGF-β-induced phospho-Smad3 expression. Data in the graph are represented as the mean ± SEM of four independent measurements. * *p* < 0.05 compared with Ad-GFP.

TGF-β/Smad signaling is a central regulator of liver fibrosis, in which Samd3 plays a major role. Phosphorylation of Smad3 and its subsequent nuclear translocation are critical steps in the signaling cascade leading to hepatic fibrosis [16,17]. Therefore, we investigated whether clusterin inhibits Smad3 phosphorylation. As expected, the levels of phosphorylated Smad3 were higher in TAA-injected mice than in control mice, but overexpression of clusterin attenuated this increase (Figure 7A). Consistent with the results of the in vivo experiments, clusterin attenuated TGF-β-induced Smad3 phosphorylation and nuclear translocation in LX2 cells (Figure 7B,C).

## 4. Discussion

The results presented here demonstrate that clusterin reduces hepatic fibrosis by inhibiting the activation of HSCs and the Smad3 pathway. 

Clusterin is a secretory protein and its expression is increased in various disease conditions such as cholestasis, liver fibrosis, hepatocellular carcinoma and renal fibrosis [18,19,20]. In our previous study, we found that clusterin expression was increased in the renal tubular area after unilateral urethral obstruction (UUO) [15]. In addition, another study found that clusterin expression was increased in the methionine and choline deficient (MCD) diet-induced steatohepatitis animal model [14], and overexpression of clusterin in this model improved liver inflammation and reduced αSMA expression in liver tissues by reducing inflammatory cytokine and oxidative stress [14]. According to previous studies, upregulation of clusterin serves as a defense mechanism to prevent disease. In our current study, we observed increased clusterin expression in cirrhotic human livers, as well as in TAA-injected and BDL mouse models. Loss of clusterin promoted hepatic fibrosis after TAA injection, whereas overexpression of clusterin significantly attenuated TAA-induced hepatic fibrosis. Overall, these findings demonstrate that clusterin functions as a defense mechanism to prevent hepatic fibrosis.

Chronic treatment of mice with TAA induces liver damage, fibrosis, and eventually cirrhosis, which is associated with HSCs [21]. In response to liver injury, HSCs are activated to transform from a quiescent state into myofibroblast-like cells [22]. Activated HSCs are αSMA-positive cells that produce extracellular matrix (ECM) proteins such as type I collagen. In fact, fibrosis is characterized by the excessive accumulation of ECM proteins and occurs in most types of chronic liver disease [23,24]. Therefore, studies investigating the direct treatment of activated HSCs as a therapy for liver fibrosis are currently in progress [25]. In our current study, clusterin expression increased during the activation of primary HSCs, and overexpression of clusterin inhibited αSMA and collagen expression in primary HSCs and LX2 cells. These data indicate that the inhibitory effect of clusterin on liver fibrosis involves a negative effect on HSC activation.

TGF-β is a major profibrogenic molecule. In general, Smad3 phosphorylated by TGF-β is translocated to the nucleus and initiates transcription of a TGF-β target gene comprising the ECM protein [17,26]. Previous studies have shown that clusterin reduces TGF-β-induced phospho-Smad3 expression in kidney cells [15]. Similarly, in our current study, clusterin inhibited TGF-β-induced phospho-Smad3 expression in LX2 cells. Furthermore, clusterin suppressed Smad3 nuclear translocation, suggesting that it also plays an important role in suppressing phospho-Smad3 expression in the liver. 

In conclusion, upregulation of clusterin in the fibrotic liver can be a protective response whatever the cause of fibrosis, and clusterin reduces hepatic fibrosis by inhibiting HSC activation and Smad3 signaling pathways.

## Supplementary Materials

The following are available online at https://www.mdpi.com/2073-4409/8/11/1442/s1. Supplementary Figure S1: Loss of clusterin increases the expression of PAI-1 after TAA injection. Supplementary Figure S2: Clusterin inhibits collagen in primary HSC. Supplementary Figure S3: Clusterin inhibits fibronectin expression in LX2 cells.
