# Supplementary material for: Clusterin Attenuates Hepatic Fibrosis by Inhibiting Hepatic Stellate Cell Activation and Downregulating the Smad3 Signaling Pathway
Source: Cells. 2019 Nov 14;8(11):1442. doi: 10.3390/cells8111442 (PMC6912488; doi:10.3390/cells8111442)
Supplement: Supplementary file 1 [file cells-08-01442-s001.pdf]

Supplementary chemicals

The anti-PAI-1 antibody (612025) and anti-fibronectin (610077) were purchased from BD bio sciences (San Jose, CA, USA)

Supplementary Figure S1. Loss of clusterin increases the expression of PAI-1 after TAA injection.

Representative western blot analysis of PAI-1 expression in the livers of wild-type (Clu+/+) and clusterin KO (Clu-/-) mice after TAA injection. Data in the graph are represented as the mean ± SEM. *P < 0.05 compared with Clu+/+.

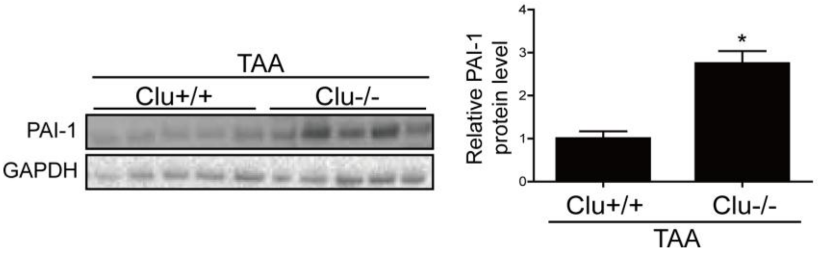

Supplementary Figure S2. Clusterin inhibits collagen in primary HSC.

Western blot analysis of collagen expression in cultured HSCs at 7 days. Primary HSCs were infected with Ad-Clu and harvested 24 h later.

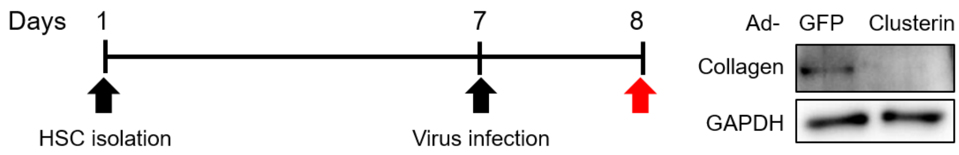

Supplementary Figure S3. Clusterin inhibits fibronectin expression in LX2 cells.

Western blot analysis showing the effect of clusterin on TGF-β-induced fibronectin expression in LX2 cells. Data in the graph are represented as the mean ± SEM of three independent measurements. *P < 0.05 compared with the control, #P < 0.05 compared with TGF-β.

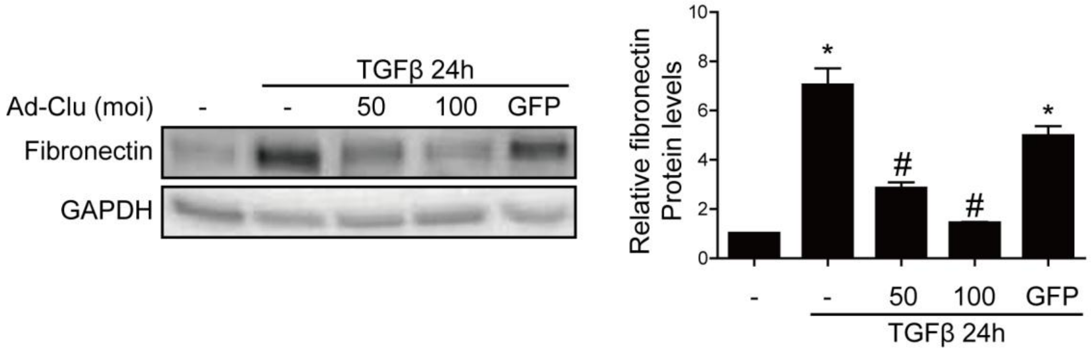
TGFβ 24h
Ad-Clu (moi)
-
-
50
100
GFP
Fibronectin
GAPDH
Relative fibronectin Protein levels
10
8
6
4
2
0
*
#
#
*
-
-
50
100
GFP
TGFβ 24h
